# Supplementary figures and images for: A machine learning approach for quantifying age-related histological changes in the mouse kidney
Source: GeroScience. 2023 Dec 16;46(2):2571–81. doi: 10.1007/s11357-023-01013-y (PMC10828469; doi:10.1007/s11357-023-01013-y)

Supplemental Figure 1

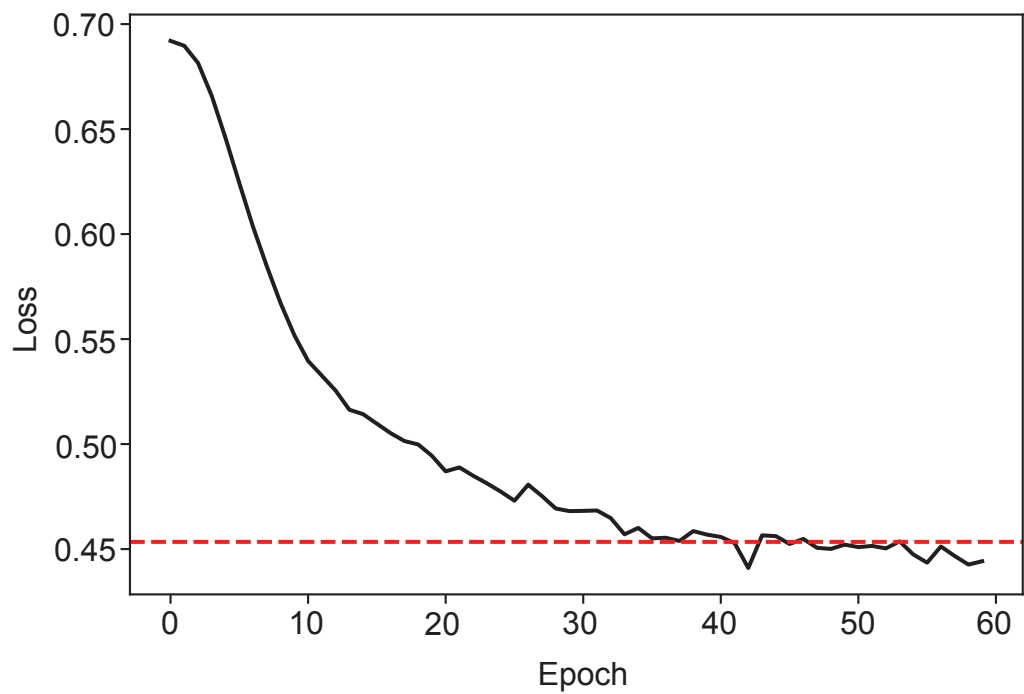

Supplement: Supplementary file 1 — Supplemental Fig. 1 This is a plot that shows the loss function on the y-axis and epoch of training on the x-axis. The dashed red line indicates the average loss value of the converged value after epoch 35. (PDF 392 KB) [file 11357_2023_1013_MOESM1_ESM.pdf]

**A**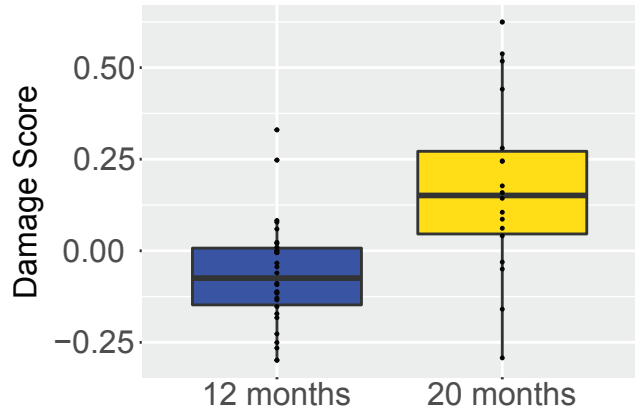**B**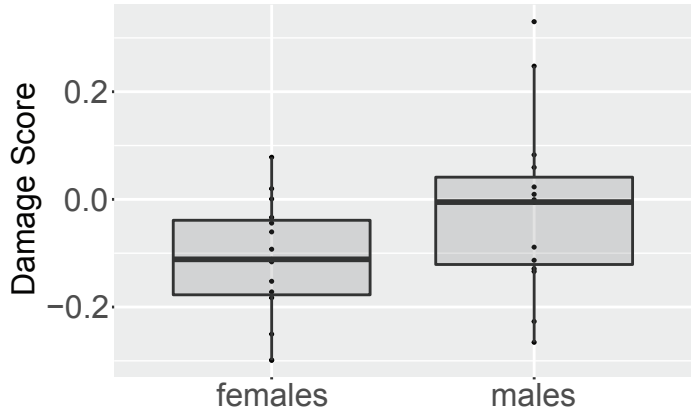

Supplement: Supplementary file 2 — Supplemental Fig. 2 This is a plot showing electronic geropathology score on the y axis the x axis shows different ages of mice (A) or different sexes of 12-month-old mice (B). Colors in panel A are for reference and match the colors in Fig. 1 (PDF 404 KB) [file 11357_2023_1013_MOESM2_ESM.pdf]
